# Glucose control in home-isolated adults with type 1 diabetes affected by COVID-19 using continuous glucose monitoring

**DOI:** 10.1007/s40618-021-01669-3

**Published:** 2021-09-05

**Authors:** M. Longo, L. Scappaticcio, M. Petrizzo, F. Castaldo, A. Sarnataro, D. Forestiere, F. Caiazzo, G. Bellastella, M. I. Maiorino, A. Capuano, K. Esposito

**Affiliations:** 1 Department of Advanced Medical and Surgical Sciences, University of Campania “Luigi Vanvitelli”, Piazza Luigi Miraglia 2, 80138 Naples, Italy; 2 Division of Endocrinology and Metabolic Diseases, University of Campania “Luigi Vanvitelli”, Piazza Luigi Miraglia 2, 80138 Naples, Italy; 3 Unit of Diabetes, University of Campania “Luigi Vanvitelli”, Naples, Italy; 4 Section of Pharmacology “L. Donatelli”, Department of Experimental Medicine, University of Campania “Luigi Vanvitelli”, Campania Regional Centre for Pharmacovigilance and Pharmacoepidemiology, Naples, Italy

**Keywords:** Type 1 diabetes, COVID-19, Glucose control, CGM, MDI, CSII

## Abstract

**Purpose:**

This study is aimed at evaluating changes in metrics of glucose control in home-isolated patients with type 1 diabetes and COVID-19 using a continuous glucose monitoring (CGM) system.

**Methods:**

We included adults aged 18–45 years with type 1 diabetes, using CGM, followed by telemedicine at a Southern Italian University Hospital. Thirty-two home-quarantined subjects with SARS-CoV-2 positive swab constituted the COVID-19 group. Thirty age-matched diabetic individuals without COVID-19 formed the control group. The effects of COVID-19 on glycemic control in patients infected were assessed at different time points [2 weeks before-COVID-19 (Time 1), 2 weeks during-COVID-19 (Time 2) and 2 weeks after COVID-19 (Time 3)] and compared with those without infection.

**Results:**

A significant reduction of TIR (Time 1 vs Time 2, %, 60.1 ± 16.6 vs 55.4 ± 19.2, *P* = 0.03), associated with a significant increase of TAR level 2 (10.1 ± 7.3 vs 16.7 ± 12.9, *P* < 0.001), GMI (7.1 ± 0.6 vs 7.5 ± 0.8, *P* < 0.001), CV (37.3 ± 7.1 vs 39.6 ± 7.0, *P* = 0.04), mean glucose values (mg/dL, 160.2 ± 26.5 vs 175.5 ± 32.6, *P* = 0.001) and standard deviation (59.2 ± 13.1 vs 68.6 ± 17.7, *P* = 0.001) was observed in patients with COVID-19. No significant change of glycemic metrics was found in the NO COVID-19 group across the time.

**Conclusion:**

Young home-isolated patients with type 1 diabetes and COVID-19 showed a worsening of glucose control during COVID-19, as compared with age-matched diabetic subjects without the infection.

## Introduction

At the end of 2019, the severe acute respiratory syndrome coronavirus 2 (SARS-CoV-2) started to emerge in China and in most countries all over the word, causing the well-known novel coronavirus disease 2019 (COVID-19) which quickly turned into a declared pandemic. Nearly 145 million cases and more than 3.5 million deaths have been reported worldwide at the end of April 2021 [1].

Much attention has been paid to the different comorbidities involving individuals infected by SARS-CoV-2 [2, 3]. Diabetes is not associated with a higher risk to contract the infection [4, 5], but worsens the prognosis of infected people [6] leading frequently to hospital admission, acute respiratory syndrome and mortality [7, 8]. A recent English whole-population analysis showed that both type 1 and type 2 diabetes were independently associated with a more than two-fold increased risk of in-hospital death with COVID-19 [9]. Moreover, hyperglycemia is associated with increased mortality in critically ill patients affected by COVID-19 [10].

Most of evidence relating COVID-19 to diabetes has mainly focused on people with type 2 diabetes with chronic vascular complications [3, 11]. Conversely, data on patients with type 1 diabetes and COVID-19 are still very limited and restricted to elderly people in the hospital setting [9]. This may be explained by the lower prevalence of type 1 than type 2 diabetes [12] and the more frequent pauci-symptomatic course of the SARS-CoV-2 infections in individuals affected by type 1 diabetes [13].

Glucose control has a crucial role in the management of diabetes in individuals affected by COVID-19. A recent study of 35 hospitalized individuals affected by COVID-19 with diabetes showed an increased risk of adverse outcomes (admission to the intensive care unit, need for mechanical ventilation, or morbidity with critical illness) in patients with glucose levels > 160 mg/dL and < 70 mg/dL and a high glucose variability detected by an intermittently continuous glucose monitoring (iCGM) [14]. On the other hand, in a retrospective study of patients with COVID-19 and pre-existing type 2 diabetes, a well-controlled diabetes with low glycemic variability was associated with reduced medical interventions, major organ injuries, and all-cause mortality [15].

Data on the management of glucose control in patients with type 1 diabetes and concurrent infection by SARS-CoV-2 not requiring hospitalization are lacking. This study aims at evaluating the changes in meters of glucose control in a cohort of patients with type 1 diabetes infected by SARS-CoV-2 using a continuous glucose monitoring (CGM) system in the phases preceding, intercurrent and following COVID-19; the same parameters have been assessed in an age-matched cohort of type 1 diabetic subjects without COVID-19 followed by telemedicine visits in the same period.

## Research design and methods

This is a retrospective case–control study of patients with type 1 diabetes followed at Diabetes Unit of the University Hospital “Luigi Vanvitelli” in Naples (Italy). We included in the study people with type 1 diabetes followed by telemedicine who underwent a real-time PCR (RT-PCR) test on nasopharyngeal swab for SARS-CoV-2 between September 2020 and January 2021. Those with a positive result and not requiring hospitalization were included in the case group (COVID-19 group). Age and sex-matched patients with type 1 diabetes and negative test were consecutively enrolled as the control group (NO COVID-19 group). According to the Italian protocol, patients with COVID-19 needed to repeat sequential nasopharyngeal swabs after at least 14 days of self-isolation at home in order to demonstrate the remission of the disease. This was a retrospective case notes analysis study and, as such, the local ethical committee was notified on data collection; however, all patients gave their consent for the use of their personal data for scientific research.

### Study participants

Men and women with type 1 diabetes were included in the study if they attended the Unit of Diabetes at the University Hospital Luigi Vanvitelli (Naples, Italy), were using real-time CGM (Dexcom G6) or iCGM (Free Style, Abbott) in conjunction with intensive insulin therapy (multiple daily insulin injection therapy, MDI, or continuous subcutaneous insulin infusion regimen, CSII) or a hybrid closed loop system (Medtronic 670G) for at least 6 months prior the study with a sensor use > 70%, were sharing data on web-based platform (Carelink, Clarity, Libreview) and were followed by telemedicine between September 2020 and January 2021. Patients would be excluded if they did not agree to be remotely connected to the Diabetes Unit, did not upload the data before the visits, used corticosteroids or high doses of ascorbic acid (vitamin C, dosage > 1000 mg/dl) during the infection.

### Clinical variables and definition of periods

Age, sex, duration of diabetes, weight, body mass index (BMI), most recent glycosylated hemoglobin (HbA1c) values, lipid profile, renal function parameters, including creatinine levels and estimated glomerular filtration rate (eGFR), the presence of micro or macrovascular complications and autoimmune diseases were collected for each patient from individual medical records and transferred into an internal medical database. During the visits, a structured questionnaire was proposed to assess the working status, tobacco use, lifestyle habits, physical activity, as well as information about the COVID-19 (times and outcomes for each nasopharyngeal swab for SARS-CoV-2, number of close relatives of patients infected, symptoms developed during the disease and the related-therapy).

Data on glycemic control were remotely extracted during the visits from the above mentioned web-based platforms, with an observation frame for each patient of 2 weeks before the first RT-PCR positive test for SARS-CoV-2 (Time 1, Pre-COVID-19), 2 weeks immediately after the first RT-PCR positive test (Time 2, During-COVID-19) and 2 weeks immediately after the first RT-PCR negative test for SARS-CoV-2 (Time 3, Post-COVID-19). For each period, all data including CGM-related metrics [mean glucose, standard deviation (SD), coefficient of variation (CV), glucose management indicator (GMI), percentage of time spent in the range of normoglycemia (TIR, 70–180 mg/dL), percentage of time spent below range (TBR, level 1 between 54 and 69 mg/dL, level 2 < 54 mg/dL) and percentage of time spent above range (TAR, level 1 between 181 and 250 mg/dL, level 2 between 251 and 400 mg/dL)] were collected. Our primary endpoint was the change in TIR, TAR level 1 and 2, TBR level 1 and 2, before, during and after the COVID-19.

### Statistical analysis

Descriptive statistics were used to characterize the study sample. The continuous variables are shown as the mean values ± standard deviation, and the categorical variables are expressed in frequencies and percentages. Repeated measure ANOVA analyses were performed for each variable, with Tukey’s test correction, when needed. Statistical significance was accepted at *P* < 0.05. All statistical analyses were performed using SPSS software (version 10.05, Chicago, IL, USA).

## Results

Sixty-seven subjects were evaluated for the inclusion in the study. After excluding 5 patients, who assumed corticosteroids, a total of 62 adults with type 1 diabetes have been included, of whom 32 patients with confirmed COVID-19 (COVID-19 group) and 30 patients without COVID-19 (NO COVID-19 group). Mean age of participants in the study was 30.1 years and mean BMI was 25.4 kg/m^2^; moreover, mean HbA1c level was of 7.1% (54 mmol/mol) with total daily insulin dose of 42.2 U/day. Fifty-seven percent of patients were females, 43% were smoking and 55% were using a CSII system; all participants were using CGM sensor > 70% of time. Moreover, 46% of patients were using an iCGM (Free Style, Abbott), 32% a real-time CGM (rt-CGM) (Dexcom G6) and 22% a hybrid closed loop system (HCLS) (Medtronic 670G). The clinical characteristics of the two groups are shown in Table 1. There were no significant differences in all the clinical variables evaluated between the two groups. However, at similar level of HbA1c, patients of COVID-19 group showed a worse TIR, TAR and glucose variability related parameters as compared with individuals of NO COVID-19 group. The proportion of patients using CGM (iCGM or rt-CGM) or HCLS was similar in both COVID-19 and NO COVID-19 groups.

**Table 1:** Baseline clinical characteristics of patients with type 1 diabetes with and without COVID-19

Variables	COVID-19 group (*n* = 32)	NO COVID-19 group (*n* = 30)	*P*
Age, years	32.4 ± 12.8	27.6 ± 9.3	0.109
Diabetes duration, years	13.4 ± 8.8	12.7 ± 8.8	0.769
Weight, kg	73.6 ± 19.0	71.5 ± 11.7	0.663
BMI, kg/m^2^	25.0 ± 6.4	25.7 ± 4.2	0.699
Male sex, *n* (%)	13 (40)	14 (46)	0.658
CSII users, *n* (%)	17 (53)	17 (56)	0.826
iCGM users, *n* (%)	16 (50)	12 (40)	0.592
rtCGM users, *n* (%)	9 (28)	11 (37)	0.655
HCLS users, *n* (%)	7 (22)	7 (23)	0.868
Smoking, % (*n*)	12 (37)	15 (50)	0.462
HbA1c, %	7.2 ± 0.6	6.9 ± 0.6	0.107
HbA1c, mmol/mol	55 ± 17	52 ± 17	0.490
Fasting plasma glucose, mg/dL	164.3 ± 25.6	156.4 ± 22.1	0.200
SBP, mmHg	122.7 ± 10.3	121.2 ± 12.4	0.605
DBP, mmHg	73.4 ± 8.9	70.6 ± 7.3	0.182
Total cholesterol, mg/dL	149.8 ± 22.7	154.2 ± 28.6	0.566
Cholesterol HDL, mg/dL	54.2 ± 7.6	51.8 ± 8.2	0.336
Cholesterol LDL, mg/dL	80.2 ± 15.9	88.7 ± 18.7	0.172
Triglycerides, mg/dL	68.3 ± 20.1	70.2 ± 19.8	0.709
Creatinine, mg/dL	0.9 ± 0.2	0.8 ± 0.1	0.108
eGFR, ml/min	100.0 ± 22.5	109.8 ± 25.0	0.204
Microvascular complications, *n* (%)	2 (6)	1 (3)	0.954
Autoimmune diseases, *n* (%)	10 (31)	12 (40)	0.650
GMI, %	7.1 ± 0.6	7.0 ± 0.4	0.446
Mean glucose, mg/dL	160.2 ± 26.5	152.0 ± 19.9	0.176
SD, mg/dL	59.2 ± 13.1	52.1 ± 9.4	0.018
CV, %	37.3 ± 7.1	33.9 ± 5.1	0.035
TIR, % (70–180 mg/dL)	60.1 ± 16.6	70.2 ± 9.3	0.005
TAR level 1, % (181–250 mg/dL)	25.8 ± 13.0	21.7 ± 8.2	0.146
TAR level 2, % (251–400 mg/dL)	10.1 ± 7.3	5.4 ± 4.7	0.004
TBR level 1, % (54–69 mg/dL)	3.8 ± 2.9	3.0 ± 3.2	0.306
TBR level 2, % (< 54 mg/dL)	1.2 ± 2.5	0.8 ± 1.5	0.452
Total daily insulin dose, U/day	43.4 ± 15.5	40.3 ± 21.9	0.520

Data are expressed as mean and standard deviation (SD)

*BMI* Body mass index, *CSII* continuous subcutaneous insulin infusion, *CV* coefficient of variation, *DBP* diastolic blood pressure, *eGFR* estimated glomerular filtration rate, *GMI* glucose management indicator, *SBP* systolic blood pressure, *HDL* high density lipoprotein, *LDL* low density lipoprotein, *SD* standard deviation, *TAR* time above range, *TBR* time below range, *TIR* time in range

More than 50% of patients contracted the infection by a direct contact (family members, partner, friends); in 44% of patients the cause of the contagion remained unknown. The infection had an asymptomatic course in 12% of cases. The reported symptoms included fever (62%), anosmia and dysgeusia (50%), body aches (37%), cold (25%), dry cough (21%), nausea and diarrhea (18%). No patient in the COVID-19 group experienced diabetic ketoacidosis. Mean recovery duration of COVID-19 until the first negative nasopharyngeal swab was 20.8 days (duration range 11–30 days). Azithromycin was the most used drug for COVID-19 (62%), followed by supplements of vitamins C (< 1000 mg/die) and D (60%) and paracetamol (22%).

Table 2 shows changes in the main glucose parameters during the 3 different time points analyzed in the two groups. At Time 2, there was a significant reduction of TIR (Time 1 vs Time 2, %, 60.1 ± 16.6 vs 55.4 ± 19.2, *P* = 0.03), associated with a significant increase in GMI (%, 7.1 ± 0.6 vs 7.5 ± 0.8, *P* < 0.001), and CV (%, 37.3 ± 7.1 vs 39.6 ± 7.0, *P* = 0.04) in the COVID-19 group, but not in the NO COVID-19 group. Moreover, a significant increase in TAR level 2 (%, 10.1 ± 7.3 vs 16.7 ± 12.9, *P* < 0.001), mean glucose values (mg/dL, 160.2 ± 26.5 vs 175.5 ± 32.6, *P* = 0.001) and SD (mg/dL, 59.2 ± 13.1 vs 68.6 ± 17.7, *P* = 0.001) were found in the COVID-19 group at Time 2, without significant changes of total insulin doses, which slightly increased only during Time 2. All the metrics, including GMI, mean glucose, SD, CV, TIR and TAR level 2, improved during Time 3 (Post-COVID-19 phase). No significant change of glycemic metrics was observed in the NO COVID-19 group across the different time points considered.

**Table 2:** Changes in glycemic metrics in COVID-19 and NO COVID-19 group in different time points

Variables	COVID-19 group (*N* = 32)				NO COVID-19 group (*N* = 30)			
	Time 1 (pre COVID-19)	Time 2 (during COVID-19)	Time 3 (post COVID-19)	*P*	Time 1	Time 2	Time	*P*
GMI, %	7.1 ± 0.6	7.5 ± 0.8*	7.2 ± 0.9	< 0.001	7.0 ± 0.4	7.1 ± 0.5	7.0 ± 0.3	0.228
Mean glucose, mg/dL	160.2 ± 26.5	175.5 ± 32.6*	168.2 ± 41.6	0.002	152.0 ± 19.9	153.8 ± 21.0	150.8 ± 16.6	0.192
SD, mg/dL	59.2 ± 13.1	68.6 ± 17.7*	63.2 ± 20.6	< 0.001	52.1 ± 9.4	54.1 ± 11.8	53.3 ± 12.6	0.342
CV, %	37.3 ± 7.1	39.6 ± 7.0*	36.6 ± 7.5	0.031	33.9 ± 5.1	34.4 ± 7.1	34.8 ± 6.8	0.554
TIR, % (70–180 mg/dL)	60.1 ± 16.6	55.4 ± 19.2*	59.1 ± 20.4	0.024	70.2 ± 9.3	69.9 ± 8.8	71.3 ± 8.9	0.202
TAR level 1, % (181–250 mg/dL)	25.8 ± 13.0	24.3 ± 9.5	22.9 ± 12.1	0.505	21.7 ± 8.2	22.2 ± 7.2	20.8 ± 8.1	0.434
TAR level 2, % (251–400 mg/dL)	10.1 ± 7.3	16.7 ± 12.9*	13.6 ± 10.8	< 0.001	5.4 ± 4.7	5.6 ± 4.4	5.1 ± 3.9	0.180
TBR level 1, % (54–69 mg/dL)	3.8 ± 2.9	2.7 ± 2.5	3.0 ± 2.6	0.071	3.0 ± 3.2	2.7 ± 3.1	3.0 ± 4.0	0.856
TBR level 2, % (< 54 mg/dL)	1.2 ± 2.5	0.8 ± 1.2	0.7 ± 1.5	0.165	0.8 ± 1.5	0.7 ± 1.4	0.5 ± 1.1	0.157
Total daily insulin dose, U/day	43.4 ± 15.5	52.4 ± 11.7	47.5 ± 10.4	0.229	40.3 ± 21.9	40.8 ± 20.6	40.8 ± 20.5	0.724

Data are expressed as mean and standard deviation

*CV* coefficient of variation, *GMI* glucose management indicator, *SD* standard deviation, *TAR* time above range, *TIR* time in range, *TBR* time below range

*P < 0.05 vs Time 1

When dividing the COVID-19 group according to the insulin regimen therapy, at Time 2 we found a corresponding significant increase in GMI (*P* < 0.001), mean glucose (*P* < 0.001), SD (*P* = 0.001), and TAR level 2 (*P* = 0.003) associated with a reduction of TBR level 2 in patients treated with MDI (*N* = 15) but not in those using CSII (*N* = 17) (Table 3). Participants with CSII did not show any significant change in metrics of glucose control except for a reduction in TAR level 1 (*P* = 0.001) and TBR level 2 (*P* = 0.051) at Time 2. Moreover, we observed a worsening of GMI and TAR level 2 in patients with and without fever (Table 4). Furthermore, those with fever showed a significant increase also in mean glucose, SD and total daily insulin dose.

**Table 3:** Changes in glucose parameters in patients of COVID-19 group according to insulin regimen therapy

Variables	MDI group (*n* = 15)				CSII group (*n* = 17)			
	Time 1 (pre COVID-19)	Time 2 (during COVID-19)	Time 3 (post COVID-19)	*P*	Time 1 (pre COVID-19)	Time 2 (during COVID-19)	Time 3 (post COVID-19)	*P*
GMI, %	6.9 ± 0.5	7.5 ± 0.7*	7.3 ± 0.8	< 0.001	7.2 ± 0.7	7.5 ± 0.8	7.3 ± 0.9	0.143
Mean glucose, mg/dL	152.1 ± 22.6	176.0 ± 31.7*	166.8 ± 34.9	< 0.001	167.3 ± 28.2	174.9 ± 34.5	169.6 ± 49.1	0.450
SD, mg/dL	56.0 ± 13.1	72.1 ± 17.9*	64.6 ± 19.7	0.001	61.9 ± 12.8	65.5 ± 17.5	61.8 ± 22.3	0.269
CV, %	37.6 ± 9.2	40.9 ± 7.6	36.9 ± 8.8	0.076	37.1 ± 5.1	38.4 ± 6.4	36.3 ± 6.3	0.383
TIR, % (70–180 mg/dL)	60.1 ± 18.2	52.6 ± 20.5	54.3 ± 18.8	0.061	60.1 ± 16.8	59.2 ± 19.3	64.7 ± 21.5	0.324
TAR level 1, % (181–250 mg/dL)	27.1 ± 16.5	27.9 ± 9.8	27.2 ± 13.6	0.862	24.6 ± 9.3	21.0 ± 8.3*	18.7 ± 8.8	0.001
TAR level 2, % (251–400 mg/dL)	7.4 ± 3.4	17.1 ± 12.8*	14.0 ± 7.0	0.003	12.4 ± 8.9	16.5 ± 13.4	13.3 ± 13.9	0.060
TBR level 1, % (54–69 mg/dL)	4.9 ± 3.4	3.1 ± 2.0	3.2 ± 2.9	0.074	2.9 ± 1.9	2.4 ± 3.2	2.9 ± 2.4	0.576
TBR level 2, % (< 54 mg/dL)	2.3 ± 3.4	0.9 ± 1.4*	0.8 ± 1.9	0.009	0.4 ± 0.5	0.7 ± 1.0	0.6 ± 1.1	0.051
Total daily insulin dose, U/day	46.6 ± 19.6	55.6 ± 18.5*	44.6 ± 15.9	0.002	40.6 ± 10.6	49.8 ± 8.5	48.7 ± 11.9	0.334

Data are expressed as mean and standard deviation

*CSII* continuous subcutaneous insulin infusion, *CV* coefficient of variation, GMI glucose management indicator, *MDI* multiple daily insulin injection, *SD* standard deviation, *TAR* time above range, *TIR* time in range, *TBR* time below range

*P < 0.05 vs Time 1

**Table 4:** Change in glucose parameters in patients of COVID-19 group divided according to the presence or not of fever

Variables	Patients with fever (*n* = 20)				Patients without fever (*n* = 12)			
	Time 1 (pre COVID-19)	Time 2 (during COVID-19)	Time 3 (post COVID-19)	*P*	Time 1 (pre COVID-19)	Time 2 (during COVID-19)	Time 3 (post COVID-19)	*P*
GMI, %	7.2 ± 0.7	7.6 ± 0.7*	7.2 ± 0.8	0.001	7.0 ± 0.6	7.4 ± 0.7*	7.1 ± 0.8	0.037
Mean glucose, mg/dL	165.2 ± 25.9	180.8 ± 32.5*	168.8 ± 41.5	0.009	156.6 ± 27.2	168.2 ± 28.8	163.0 ± 36.9	0.064
SD, mg/dL	62.9 ± 11.8	73.9 ± 14.9*	68.8 ± 18.5	0.009	52.4 ± 12.9	59.2 ± 16.4	50.2 ± 15.4	0.051
CV, %	38.9 ± 7.7	42.0 ± 6.2	39.7 ± 5.9	0.060	33.9 ± 3.1	34.7 ± 4.9	33.2 ± 9.1	0.760
TIR, % (70–180 mg/dL)	55.8 ± 16.4	51.4 ± 18.5*	58.5 ± 18.1	0.004	66.0 ± 15.6	61.5 ± 19.0	64.4 ± 22.0	0.233
TAR level 1, % (181–250 mg/dL)	28.1 ± 13.9	24.6 ± 9.2	22.7 ± 8.8	0.059	22.8 ± 8.8	25.6 ± 10.1	21.6 ± 15.1	0.530
TAR level 2, % (251–400 mg/dL)	11.6 ± 6.5	19.6 ± 13.1*	14.3 ± 11.3	0.001	8.6 ± 8.8	11.6 ± 9.7*	10.0 ± 9.8	0.006
TBR level 1, % (54–69 mg/dL)	4.3 ± 3.1	3.4 ± 3.0	3.6 ± 2.4	0.216	2.3 ± 1.8	1.1 ± 0.9	2.2 ± 2.2	0.300
TBR level 2, % (< 54 mg/dL)	1.7 ± 2.9	1.2 ± 1.3	1.1 ± 1.7	0.209	0.2 ± 0.4	0.1 ± 0.3	0.1 ± 0.2	0.146
Total daily insulin dose, U/day	41.0 ± 11.7	54.9 ± 12.9*	49.7 ± 11.7	0.015	43.8 ± 14.2	48.5 ± 13.9	46.2 ± 12.1	0.791

Data are expressed as mean and standard deviation

*CV* coefficient of variation, *GMI* glucose management indicator, *SD* standard deviation, *TAR* time above range, *TIR* time in range, *TBR* time below range

*P < 0.05 vs Time 1

## Conclusions

To the best of our knowledge, this is the first study reporting the changes of the metrics of glucose control during different phases of COVID-19 course in young adults with type 1 diabetes infected by SARS-CoV-2. The main characteristics of this study refer to 1) the inclusion of type 1 diabetic people not requiring hospitalization and hence placed in quarantine and 2) the remote monitoring of the patients using a CGM device. The study demonstrated a deterioration of glucose control and glucose variability in this selected population, as demonstrated by the significant increase of GMI, mean glucose, SD and time spent above 250 mg/dl. This trend was more pronounced in patients treated with MDI therapy than those using CSII. Interestingly, the worsening of glucose control was independent of the presence of fever, as patients with and without fever showed an increase of GMI and TAR level 2.

The worse glucose control expressed by lower TIR and higher TAR and glucose variability-indices presented by patients in COVID-19 group at Time 1 may be related to both the incubation and the development of the infection, thus suggesting a potential bidirectional relationship between viral infection and glucose control. Whether poor or suboptimal glycemic control may represent a risk factor for infection for SARS-CoV-2 in patients with diabetes is still unknown; however, hyperglycemia has been extensively associated with the severity and mortality for COVID-19 [10].

There is few evidence reporting the trend of glucose control in people with type 1 diabetes affected by SARS-CoV-2, mostly coming from hospitalized adults affected by multiple comorbidities [16]. The monitoring of glucose levels represents a crucial aspect in patients affected by COVID-19, as a poor glucose control has been associated with worse outcomes in hospitalized patients with both type 1 and type 2 diabetes [16–18]. Moreover, few data on people with diabetes and COVID-19 using CGM are currently available. In a retrospective study of 562 individuals with and without diabetes, critically ill patients affected by COVID-19 spent significantly less time in the normal range of glucose levels (70–150 mg/dL) than non-COVID-19 individuals (44.4% vs. 68.5%), with a higher need of daily insulin dose [19]. Moreover, lower levels of TIR correlated with an increased risk of ventilator requirement and length [19]. In 35 hospitalized patients with diabetes using an iCGM, both TBR (time spent below 70 mg/dL) and TAR (time spent above 160–200 mg/dL) were significantly associated with composite adverse outcomes and prolonged hospitalization for an average period of 10.2 days [14]. Moreover, in a Chinese pilot study [20], 6 days-CGM data of 13 non-diabetic patients with mild symptoms of COVID-19 were compared with those coming from 3 days-CGM use in a group of 18 healthy age-matched individuals. Patients with COVID-19 had more frequently post-prandial hyperglycemia and higher glucose variability as compared with healthy subjects (CV, 25.6% vs. 15.7%, *P* < 0.001), with significantly lower TIR (time spent in the range of 70–140 mg/dL, median 80.1% vs. 93.1%, *P* = 0.001) and higher TAR (time spent above 140 mg/dL, median 13.9% vs. 2.3%, *P* = 0.006) than healthy individuals [20].

Specific viral-induced mechanisms of damage might have contributed to the worsening of glycemic control in our population, as SARS-CoV-2 seems to compromise the hepatic synthesis of glycogen and pancreatic β-cells insulin delivery [21, 22]. Moreover, the infection is associated with a huge production of cytokines which promotes the onset of stress-related conditions and inflammatory status [22]. All these events may impair glucose homeostasis and further enhance insulin resistance. Finally, self-home isolation may have worsened daily routine habits and increased physical and psychological stress induced by the infection.

The use of technologies plays a crucial role in diabetes management and is emphasized in the COVID-19 era, as testified by the fair number of studies evaluating worldwide the change of glucose control parameters in patients with type 1 diabetes during the lockdown restrictions. Despite the changes in lifestyle habits and the slowing down of daily routine, a stable or even improved glycemic control was found in patients of all ages with diabetes using a CGM or a hybrid closed loop system during the COVID-19 lockdown [23–25]. In our study, people treated with CSII therapy showed a better control of hyperglycemia and glucose variability than those using MDI therapy, even during viral infection. This finding confirms evidence coming from previous prospective studies which demonstrated that the use of CSII is more effective than MDI therapy in lowering glucose variability, fasting glycemia, and overall hypoglycemic events among young adults with type 1 diabetes [26].

Although our patients experienced a worsening of glucose control during COVID-19 phase, a slight restoration of glucose levels was immediately reached in the post-COVID-19 phase. This may coincide with the end of viral infection and the appropriate use of CGM, which helped patients to detect hyperglycemia and prevent further deteriorations of glucose control.

The limitations of the present study include the relative small number of people included, the selection of patients from a single diabetic center and the retrospective design of the study. On the other hand, the use of a valid tool (CGM) to measure metrics of glucose control, the inclusion of a cohort of young patients infected by SARS-CoV-2, the comparison of CGM data before, during and after COVID-19 course and the presence of a healthy control group represent the main strength of this study.

In conclusion, young patients with type 1 diabetes and COVID-19 treated in a home-isolation context showed a worsening of glucose control, as compared with age-matched diabetic subjected without infection. Attention should be paid to these young patients in order to prevent adverse health outcomes.

## Data Availability

All data generated or analysed during this study are included in this published article.
